# A sensitive method for the recovery of *Escherichia coli* serogroup O55 including Shiga toxin‐producing variants for potential use in outbreaks

**DOI:** 10.1111/jam.14345

**Published:** 2019-07-07

**Authors:** M. Kirchner, E. Sayers, S. Cawthraw, N. Duggett, R. Gosling, C. Jenkins, T.J. Dallman, D. Mueller‐Doblies, M.F. Anjum

**Affiliations:** ^1^ Department of Bacteriology, Animal and Plant Health Agency Addlestone Surrey UK; ^2^ University of East Anglia/Quadram Institute Bioscience, Norwich Research Park Norwich UK; ^3^ Public Health England London UK

**Keywords:** enterohaemorrhagic *Escherichia coli*, environmental, immuno‐magentic separation, pathogen, rapid diagnostics

## Abstract

**Aim:**

Shiga toxin‐producing *Escherichia coli* (STEC) cause bloody diarrhoea, kidney failure and occasionally death. However, identifying the source of infection caused by STEC other than serogroup O157 is hampered by the availability of sensitive methods for detecting these pathogens. In this study, we developed novel tools for detecting *E. coli* O55 that is potentially associated with human outbreaks.

**Methods and Results:**

Overall specificity of immuno‐magnetic separation (IMS) beads coated with anti‐O55 serum was good with exception of cross‐reactivity with *E. coli* O22 and O23, which was eliminated using an O55‐specific PCR. Limit of detection for *E. coli* O55 using O55‐IMS beads in spiked cattle faeces was on average 50 CFU per ml (range 1–90), and improved to <10 CFU per ml using the O55‐specific PCR, following IMS on samples enriched for 2 h with *E. coli* O55. Application of these tools to test cattle faeces collected on‐farm allowed the isolation of O55:H19, which through whole genome sequencing was compared to STEC O55:H7 human outbreak strains.

**Conclusion:**

These tools provide a sensitive method which could be used to screen samples for STEC O55, whether environmental or human clinical.

**Significance and Impact of the Study:**

Several human outbreaks reported in England were caused by STEC O55:H7. Tools developed here could assist in identification of the environmental source for these isolates, which has not yet been established.

## Introduction

*Escherichia coli*, although a constituent of human and animal gut microbiota can also cause intestinal and extra‐intestinal infections following the acquisition of virulence factors. Shiga toxin‐producing *E. coli* (STEC) is a pathotype that has acquired a number of virulence factors including Shiga toxins. Shiga toxins are encoded by the *stx*1 (variants a, c and d) and *stx*2 (variants a, b, c, d, e, f and g) genes and are carried by bacteriophage (Kruger and Lucchesi, 2015). STEC are important human pathogens which are sometimes acquired through the consumption of contaminated food‐stuffs or close animal contacts (Aktan *et al.* 2007; Carter *et al.* 2008; Ihekweazu *et al.* 2012; Anjum *et al.* 2014; Jenkins *et al.* 2015; Adams *et al.* 2016). STEC can cause life‐threatening infections which can include haemolytic uraemic syndrome (HUS) and haemorrhagic colitis. *E. coli* O157 is the most common STEC serogroup associated with human infection, although there are many more *E. coli* serogroups which can cause STEC infections, with serogroups O111, O145, O26, O103, O121 and O45 are regarded as the most common. These are considered the major cause of non‐O157 STEC infections (Bosilevac and Koohmaraie, 2012; Eichhorn *et al.* 2015).

In 2014–2015, an outbreak of STEC occurred in the county of Dorset, England caused by STEC serotype O55:H7 carrying *stx*2 and *ea*e, encoding an adhesin present in the locus of enterocyte effacement. In total, 31 cases of STEC O55 infection were reported mainly in children, 13 of which developed HUS (Mcfarland *et al.* 2017). The serotype O55:H7 had not previously been associated with STEC infections in England and the source of STEC O55:H7 isolates causing infections in Dorset could not be determined despite extensive investigations (Paz, 2016). Moreover, STEC O55 was reported in the county of Surrey, England, in November 2017, where it was confirmed in two people and suspected in a further five; STEC O55 from this outbreak was very closely related to the Dorset outbreak strain, belonging to the same 10 single‐nucleotide polymorphism (SNP) single linkage cluster as the Dorset strain (Brock, 2017). Outside the UK, sporadic cases of human infection with STEC O55 have been reported in Germany, Italy and the Czech republic (Johnson *et al.* 2006). In October 2018, environmental STEC O55:H7 was again identified as the causative organism in two children from Leicestershire who developed HUS, which resulted in fatality, and again the environmental source was not identified (https://www.bbc.co.uk/news/uk-england-leicestershire-45838401; Public Health England, unpublished data).

Immuno‐magnetic separation (IMS) can be used for the isolation of *E. coli* of interest, which may be present in low concentrations, in a sample contaminated with many other bacteria including other *E. coli* serotypes. Commercially produced magnetic beads are available for O157 and the six most common non‐O157 STEC serogroups (Noll *et al.* 2016), which can be used for their isolation from food, faeces, water and other environmental samples, where they may be present in low numbers (Lejeune *et al.* 2001; Islam *et al.* 2006; Guy *et al.* 2014; Kanki *et al.* 2014) (Dynabead product information, https://www.thermofisher.com). The IMS method for the detection of O157 is well established and includes an IMS step followed by plating on selective agar, specifically cefixime‐tellurite sorbitol MacConkey agar (CT‐SMAC). There are no selective agars for non‐O157 STEC making differentiation of non‐O157 STEC from other *E. coli* difficult.

There is currently no method for the selective isolation of *E. coli* O55 from faecal (human or animal) and environmental samples, which may aid in increasing the sensitivity of detection and source attribution. Therefore the aim of this study was to design and evaluate a rapid and sensitive IMS‐based method for the selection of *E. coli* O55, which could be used to screen samples for this serogroup.

## Materials and Methods

### Bead preparation

Tosylactivated M‐280 Dynabeads (ThermoFisher Scientific, Basingstoke, UK) were coated with antiserum for the *E. coli* O55 lipopolysaccharide (BioRad, Hercules, CA, USA) according to published methods (https://tools.thermofisher.com/content/sfs/manuals/dynabeads_m280tosylactivated_man.pdf). Washed beads were coated with O55 antiserum for 14 h at 37°C in 0·04 mol l^−1 ^borate buffer pH 9·5, 1·2 mol l^−1^ ammonium sulphate. Following overnight coupling, beads were washed in phosphate‐buffered saline (PBS)+0·5% bovine serum albumin (BSA), followed by two further washes in PBS + 0·1% BSA.

### Immuno‐magnetic separation

Immuno‐magnetic separation was performed using a manual method except for STEC isolation, which used an automated system. A 1 ml sample (overnight broth culture or spiked faeces suspended in BPW) was mixed with 20 µl of coated beads and incubated for 30 min at room temperature. A magnet was used to separate the beads, which were then washed in triplicate with 1000 µl PBS‐0·05% Tween before being re‐suspended in 100 µl of PBS or water. Beads were plated onto CHROMagar ECC (BioConnections, UK), a chromogenic agar on which most *E. coli* grow as blue colonies. An automated system was used to perform IMS of STEC isolates. As described above, a sample was mixed with beads and IMS performed using the Bead‐Retriever (Invitrogen Dynal, Carlsbad, CA). Following washing in PBS‐0·05% Tween, beads were plated on to CHROMagar O157 (BioConnections, Knypersley, UK). The *E.coli*/VTEC program pre‐installed on the Bead‐Retriever (Dynal) was used for IMS.

### Testing the specificity and sensitivity of O55 IMS beads

The specificity of O55 IMS beads was tested with a panel of 155 *E. coli* with different O‐antigens. Serogroups not tested were as follows: O31, O39, O47, O63, O64, O67, O68, O72, O84, O94, O122, O138, O139, O141, O146, O161, O171 and O172, as they were unavailable. Overnight cultures of 151 *E. coli* were diluted to 10^3^ CFU per ml in PBS and mixed in pools of four or five strains. Triplicate 1 ml aliquots of each pool were subjected to IMS using O55‐beads and the beads plated on LB agar. Four serogroups, O22, O23, O83 and O104, were tested singularly for cross‐reactivity with the O55‐beads. Serial dilutions of overnight cultures grown in LB broth were prepared to achieve concentrations of 10^2^ and 10^3^ CFU per ml. These were subjected to manual IMS with O55‐beads. Percentage recovery was determined following enumeration of pre‐ and post‐IMS samples plated on LB agar.

Seven *stx*‐negative strains of *E. coli* serogroup O55 (Table 1) were also tested to determine the recovery rate of cells following IMS as described above. For STEC O55, overnight cultures were prepared in LB broth and a 10‐fold dilution series prepared down to 10^−7^. IMS using O55‐beads was performed on 1 ml of dilutions 10^−5^, 10^−6^ and 10^−7^ and beads plated onto CHROMagar O157 to determine cell counts.

**Table 1:** Details of O55 strains used in this study. The origin and year of isolation are unknown for B2884 as this is a type strain from the Staten Serum Institute collection (http://www.ssidiagnostica.com/da/Products/Bacterial-strains/E-coli)

Strain ID	Year of isolation	Origin	Stx present	Serotype
B1362	2000	Bovine	Negative	O55:K+:−
B2884	Unknown	Unknown	Negative	O55:H−
165/13	2013	Bovine	Negative	O55:ND:ND
394/10	2010	Bovine	Negative	O55:ND:ND
328/11	2011	Bovine	Negative	O55:ND:ND
329/11	2011	Bovine	Negative	O55:ND:ND
384/10	2010	Bovine	Negative	O55:ND:ND
H1	2015	Human	Positive	O55:H51
H2	2016	Human	Positive	O55:H7
H3	2016	Human	Positive	O55:H12

ND, not determined.

### Limit of detection in spiked faeces

The *E. coli* O55 strain (B1362) was grown overnight at 37°C in LB broth and this was used to prepare dilutions to achieve a final concentration of between 10^7^ and 10^1^ CFU per ml. One gram of fresh cattle faeces (1–2 days old) was mixed with 9 ml BPW and to this, 1 ml of O55 cell suspension (at concentration between 10^7^ and 10^1^ CFU per ml) was added; a control sample (1 g of faeces mixed with 9 ml BPW) was spiked with PBS only. One millilitre aliquots were taken from the spiked and unspiked faeces prior to the enrichment, and after 2 h incubation at 37°C. IMS was performed with O55‐dynabeads according to the method above. IMS beads were suspended in 100 µl of PBS and half of this plated onto CHROMagar ECC. Plates were incubated for 14–16 h at 37°C and blue colonies counted as presumptive *E. coli*. Latex agglutination was performed on three blue colonies from positive plates as described below. The method was considered positive for detecting *E. coli* O55 when a third of the colonies selected from plates for agglutination were positive. A cell count was performed to confirm the inoculum size, using the Miles Misra method on CHROMagar ECC (Miles *et al.* 1938). The experiment was performed in triplicate.

### Latex agglutination testing of colonies recovered following IMS

Suspected isolates of *E. coli* O55 recovered following IMS were tested by latex agglutination. A colony from selective plates was mixed with 10 µl of saline on a reaction card (http://www.ssidiagnostica.com/da/Products/Antisera/E-coli-antisera/E-coli-latex-test) before the addition of one drop of O55 ImmuLex latex agglutination sera (as above). The reagents were thoroughly mixed and following 10 min incubation at room temperature the results were read.

### Real‐time PCR detection of O55 E. coli and virulence factors

IMS‐PCR was used to confirm that samples recovered following IMS were positive for *E. coli* O55. A real‐time PCR (RT‐PCR) developed previously (DebRoy *et al.* 2005) was used to detect O55 *wzy* gene. DNA was prepared by the lysis of 50 µl of beads (post‐IMS) using heating to 99°C for 15 min. Once boiled, beads were removed and the supernatant used as a source of DNA. RT‐PCR reactions were prepared as follows: each 20 µl reaction consisted of 10 µl QuantiTect multiplex PCR master mix (Qiagen, Hilden, Germany), 10 µmol of each primer, 4 µmol of probe, water and 2 µl of DNA. PCR consisted of: 95°C for 15 min followed by 45 cycles of 95°C for 15 s and 60°C for 60 s. Fluorescence intensity was measured at the end of each amplification cycle and the cycle threshold (Ct) value was determined.

### Isolation of O55 E. coli from naturally contaminated cattle faeces

Fifty cattle faecal samples collected from a previous on‐farm investigation were screened for the presence of *E. coli* O55 (Treacy *et al.* 2019). One gram of faeces was added to 9 ml BPW and grown for 2 h at 37°C. Following incubation, a 0·5 ml sample was boiled for 15 min. PCR was performed to detect the O55 *wzy* gene and IMS performed on PCR‐positive samples using the O55‐specific IMS beads. Up to 10 blue colonies from CHROMagar ECC were tested by agglutination for O55 antigen, and positive samples prepared for DNA sequencing. Fresh growth of *E. coli* O55 on CHROMagar ECC was re‐suspended in 200 µl of water, boiled for 15 min and DNA was extracted from the lysate using the MagMAX CORE nucleic acid purification kit (ThermoFisher Scientific) and a KingFisher duo prime.

Genomic DNA was sequenced using a MiSeq v2 kit with 2x150 bp, 300 cycles (Illumina, San Diego, CA). A core genome SNP alignment was produced by Snippy (https://github.com/tseemann/snippy) using *E. coli* str. K‐12 sub‐strain MG1655 (NC_000913·3) as a reference. RAxML (https://academic.oup.com/bioinformatics/article/30/9/1312/238053) was used to build the phylogenetic tree, which was subsequently annotated using ITOL (https://www.ncbi.nlm.nih.gov/pubmed/27095192). Virulence gene carriage was assessed using APHA in‐house SeqFinder tool and an in‐house virulence gene database (Duggett *et al.* 2017).

## Results

### Determining the sensitivity and specificity of O55‐beads

**Figure 1:**
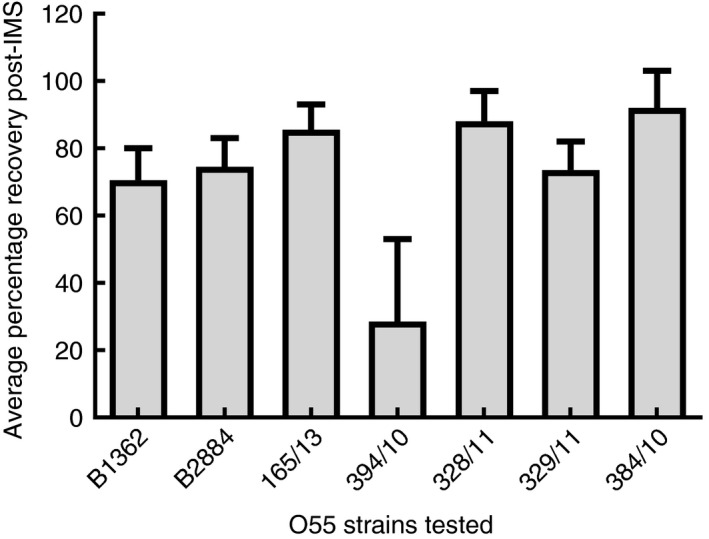
Average percentage recovery of O55 strains post‐IMS with O55‐beads. Error bars indicate the calculate standard error from mean.

The sensitivity of O55‐beads for the recovery of *E. coli* O55 isolates was tested with a panel of seven Stx‐negative *E. coli* O55 (Fig. 1). For each isolate of Stx‐negative *E. coli* O55, the recovery rate was determined at an initial inoculum of 10^2^ CFU per ml. Recovery rates were on average between 30 and 90% for Stx‐negative *E. coli* O55 with the lowest recovery rate seen for strain 394/10, indicating a natural variability in recovery between strains (Fig. 1). With the exception of strain 394/10, the recovery rate was >60%. The sensitivity of O55 beads was also tested with three strains of STEC O55. These strains included a STEC O55 isolate from the Dorset outbreak (H2) (Paz, 2016). For all three STEC tested, it was possible to recover *E. coli* O55 following IMS at a concentration of 10^2^ CFU per ml. At this concentration, an average of 1·7 blue colonies (range 1–5 colonies per plate) grew on chromogenic agar, post‐IMS.

The specificity of O55‐IMS beads was also tested with 155 *E. coli* of different non‐O55 serogroups to test their cross‐reactivity with O55‐beads. Pools of four to five different serogroups were tested at a bacterial concentration of 10^2^ CFU per ml. For each pool, the number of colonies recovered from plating of O55‐IMS elute was determined and the percentage bacterial recovery calculated. For the 31 pools tested, comprising 151 serogroups (as four serogroups were tested individually), the recovery of *E. coli* was low with only ~1·95% (range 0·06–5·29%; data not shown) recovered.

**Figure 2:**
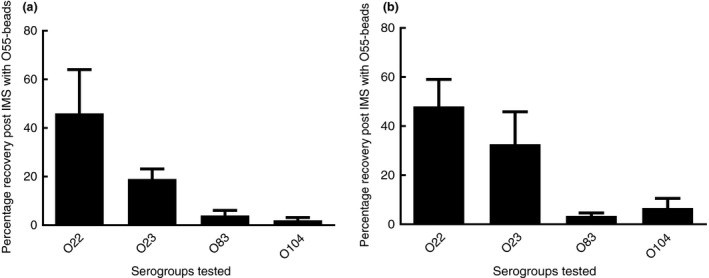
Average percentage recovery of O22, O23, O83, O104 post‐IMS with O55‐beads at different initial cell concentrations ((a) 10^2^ CFU per ml and (b) 10^3^ CFU per ml). Error bars indicate the calculated standard error from mean.

However, some strains showed higher recovery probably due to cross‐reactivity between anti‐O55 sera and these serogroups (DebRoy *et al.* 2005), and were tested individually; these included *E. coli* of serogroups O22, O23, O83 and O104. One isolate of each serogroup was tested at 10^2^ and 10^3^ CFU per ml, and the rate of recovery of isolates from suspensions post‐IMS treatment determined (Fig. 2a,b). Serogroups O83 and O104 gave low rates of recovery of *E. coli* post‐IMS. At a 10^3^ CFU per ml inoculum, the average recovery of *E. coli* O83 was 3·4 ± 1·2% and for O104 this was 6·6 ± 3·9%. Much higher rates of *E. coli* recovery were seen for serogroups O22 and O23 at 10^2^ and 10^3^ CFU per ml, which was between ~46 and 48% for serogroup O22 and ~19–33% for serogroup O23 (Fig. 2).

Due to the cross‐reactivity seen with these serogroups, an O55‐specific PCR was used to provide a method which improved the specificity of testing for *E. coli* O55 recovered via IMS. The results showed *E. coli* O22 and O23 isolates recovered through IMS were negative when using the O55‐specific PCR, while O55 isolates were positive (data not shown).

### Sensitivity of O55‐beads for recovery of O55 E. coli from faecal enrichments

The sensitivity of O55‐beads for the recovery of *E. coli* O55 from spiked faecal samples was determined. Experiments were performed with cattle faeces (1–2 days old), which were spiked with *E. coli* O55. For each spiking study, a comparison was made between IMS‐culture and IMS‐PCR, with and without a 2‐h enrichment. The limit of detection for IMS‐culture were comparable for samples not enriched (Table 2), with an average detection limit of 52 CFU per ml, compared to 54·4 CFU per ml following enrichment. For samples eluted by IMS, on which O55 PCR was performed (IMS‐PCR), the limit of detection was on average 52 CFU per ml prior to enrichment, which improved to 4·9 CFU per ml following 2‐h enrichment. The average Ct values for RT‐PCR increased as the concentration of spiked *E. coli* O55 decreased to between 22·82 (at 10^6^ CFU per ml) and 36·99 (at 10 CFU per ml), for enriched samples. Control samples, spiked with PBS, were all negative by IMS‐PCR in pre‐ and post‐enriched samples (data not shown). Also in the absence of an IMS step, PCR directly from the enrichment broths gave higher Ct values than IMS‐PCR (data not shown). In contrast, using IMS‐culture method, an average of 98·6 presumptive *E. coli* (which were blue on CHROMagar ECC) (range 40–138) were recovered from control faecal samples pre‐enrichment. Following 2‐h enrichment an average of 604 presumptive *E. coli* (range 368–1000) were recovered. Latex agglutination of presumptive *E. coli* from control samples (three blue colonies/sample) found that none of those tested were serogroup O55, indicating cross‐reaction with other O‐antigens.

**Table 2:** Limit of detection for IMS‐culture and IMS‐PCR from spiked cattle faeces. The spiking concentration at which IMS‐culture or IMS‐PCR could detect O55. The results represent the average of triplicate experiments

Enrichment time	Limit of detection (CFU per ml)	
	IMS‐culture	IMS‐PCR
O	52 (range 1–90)	52 (range 1–90)
2 h	54·4 (range 7·2–90)	4·9 (range 1–7·2)

### Analysis of naturally contaminated cattle faeces

**Figure 3:**
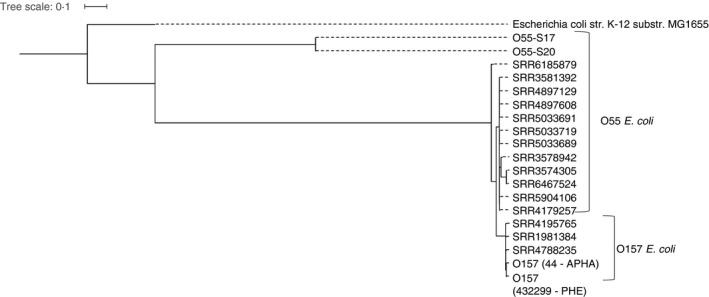
Tree generated using the SNP distance of core genomes from the isolates collected in this study (O55‐S17 and O55‐S20), *Escherichia* *coli* O55 available in BioProjects (NCBI), and O157:H7 strains (44‐APHA and 432299‐PHE) collected during a recent outbreak investigation on this farm, and representatives of O157:H7 lineages described elsewhere (SRR4788235, SRR1981384, SRR4195765) (Schutz *et al.* 2017). The *E. coli* K‐12 genome from MG1655 was used for reference.

Using the IMS‐PCR method, followed by IMS‐culture, *E. coli* O55 were isolated from two of the 50 cattle samples collected from a dairy farm, and whole genome sequence (WGS) analysis was performed on these *E. coli.* WGS data were analysed using a number of methods. Both isolates were confirmed as serotype O55:H19 using serotype finder (DTU Centre of Genomic epidemiology; http://www.genomicepidemiology.org/). Analysis of the core genomes for both isolates determined that there were 192 SNP differences between isolates. A phylogenetic tree was generated from the core genomes of the two cattle isolates of *E. coli* O55, 12 sequences of *E. coli* O55 available on NCBI BioProjects (https://www.ncbi.nlm.nih.gov/bioproject/), four of which were clinical outbreak strains, and five O157:H7 isolates, including two from cattle on this farm (Fig. 3). The cattle O55:H19 isolates were distinct from all *E. coli* O55 and O157 used for comparison and context, with >40 000 SNP differences in the core genome between the O55:H19 isolated during this study and all other isolates included in the tree. Analysis of virulence gene carriage indicated the two O55:H19 isolates lacked the virulence factors associated with STEC (such as *stx* and *eae*) and other *E. coli* pathogroups, but they carried haemolysin genes *hly*E and *hly*C, which are widely distributed on the chromosome of *E. coli* (Murase *et al.* 2012).

## Discussion

Currently, there are no protocols for the selective enrichment of *E. coli* O55 from human or animal faecal specimens. Such protocols are required to assist in the rapid identification of sources for STEC O55, especially during outbreaks. IMS, also commonly used for isolation of STEC O157, provides a sensitive isolation method when combined with selective agar. In this study, an assessment of the specificity of O55‐IMS beads produced in‐house for the isolation of STEC O55 was made with a panel of *E. coli*. It identified a low level of nonspecific binding with cross‐reaction between O55‐beads and *E. coli* of serogroups O23 and O22, which has been previously reported (DebRoy *et al.* 2005). It is not uncommon for nonspecific binding of other *E. coli* serogroups to IMS beads; cross‐reaction between beads for O111 and *E. coli* O103 has been reported (Bai *et al.* 2012).

An enrichment step is used for the isolation of STEC serotypes such as O157:H7 and O26, as they may be present in low numbers, and was trialled in this study to assist in the isolation of *E. coli* O55 from cattle faeces. However, the inclusion of an enrichment step resulted in high‐cell densities, which inhibited binding of the target *E. coli* to IMS beads (Hallewell *et al.* 2017). Conrad *et al. *(2014) found the identification of single colonies of the correct serogroup when identifying O26, O45, O103, O111, O121 and O145 on isolation plates post‐IMS to be very difficult, due to high background contamination (Conrad *et al.* 2014); but selective agar media such as CT‐SMAC for O157 increases the likelihood of detection. Currently, there are no such selective agars for STEC O55 and most other non‐O157 STEC serogroups, therefore, for our studies high background interfered with the ability to identify colonies of *E. coli* O55. Further work is required to identify an O55‐specific metabolic marker, which could be exploited to develop a novel isolation agar.

It has been demonstrated in many studies that the use of PCR provides a robust and sensitive method to screen samples for STEC. The inclusion of a PCR step in our assay improved the specificity and sensitivity of *E. coli* O55 identification. Importantly, background levels of non‐O55 *E. coli* did not affect the limit of detection for IMS‐PCR. IMS‐PCR on samples that had been enriched for 2 h was able to detect O55 spiked in faeces at concentration of <10 CFU per ml. The infectious dose of STEC O157:H7 is thought to be less than 100 CFU (Smith *et al.* 2014). The infectious dose for STEC O55 is unknown but if we assume a similar dose is required, our IMS‐PCR method is sensitive enough to detect below this level. However, the draw‐backs of using PCR alone is the lack of an O55 isolate for further characterization, as PCR does not demonstrate viability of the *E. coli*, and it does not directly link the virulence factors (*stx*) to the serogroup and isolate. Nevertheless, as implemented in this study, the PCR element of an IMS‐PCR procedure provided a robust screening tool to identify positive samples, while plating a portion of the IMS‐eluted beads on agar plates provided colonies for further analysis.

The tools developed in this study were also used to screen cattle faeces collected from a farm linked to an outbreak of STEC O157, for the presence of *E. coli* O55. Cattle faeces were screened by IMS‐PCR to determine if STEC O55 may simultaneously be present on farm. Two *E. coli* O55:H19 were successfully isolated from cattle faeces, which lacked the virulence factors such as *stx*2 and *eae* and were shown by WGS analysis to be distinct from the strain of *E. coli* O55:H7 that caused the Dorset outbreak (Schutz *et al.* 2017). It is unknown how common *E. coli* O55 is in cattle as researchers seldom examine animal faeces for the presence of commensal *E. coli* of a particular serogroup. In the UK, *E. coli* O55 (non‐STEC) has previously been isolated from cattle faeces; however, this was during an investigation of antimicrobial resistance on a dairy farm and the *E. coli* O55 was selected because it had a particular resistance trait (Horton *et al.* 2016). Furthermore, analysis of clinical submissions from diseased animals in the UK did not identify any STEC O55 isolates between 2005–2008 (Hutchinson *et al.* 2011). In that study, the predominant STEC serogroup in cattle was O26. However, STEC O55 have been detected in cattle samples in other countries including surveys of beef cattle (faeces) in Japan (Mekata *et al.* 2014), and in Ireland STEC O55 has been reported from beef carcasses (Monaghan *et al.* 2012).

Core genome analysis demonstrated the two O55:H19 were distinct from O157:H7 and O55:H7 outbreak isolates, which were more similar to each other. Phylogenetic analysis of STEC O157:H7 and STEC O55:H7 has indicated that they have a common progenitor, and O157:H7 evolved through acquisition of the Shiga toxin genes (Schutz *et al.* 2017). Our analysis suggested that other non‐STEC O55 which are unrelated to the outbreak clone may be present in the cattle faeces. Notably, these results indicate that the O55 IMS‐PCR method developed in this study is a sensitive and specific tool that can be used to identify the presence of *E. coli* O55 in faecal specimens. The isolate can be purified subsequently by IMS‐culture and characterized using other molecular methods such as WGS.

Therefore the use of IMS O55‐beads on enriched samples, followed by PCR is a method for selection of *E. coli* O55 from contaminated samples, especially those from cattle faeces where *E. coli* O55 may be present at low levels. In the future, the use of these tools to examine other samples such as environmental samples could be explored, to help support investigation of human outbreaks of infection with STEC O55. The low infectious dose characteristic of STEC makes the need for detecting low‐level contamination a key component of any STEC assay. Due to the lack of selective agars for the isolation of non‐O157 STEC, in future, consideration will be given to the evaluation of selective media for the differentiation and recovery of O55‐STEC from culture. Optimization of enrichment could also be considered as the presence of high levels of nontarget *E. coli* can interfere with binding of target *E. coli*, which could be influenced by enrichment media, temperature and time. In conclusion the tools developed in this study could be used in future for rapid analysis of human, animal or environmental samples during outbreak investigations.

## Conflict of Interest

No conflict of interest declared.
